# Catch the wave: Metabolomic analyses in human pathogenic fungi

**DOI:** 10.1371/journal.ppat.1008757

**Published:** 2020-08-20

**Authors:** Philipp Brandt, Enrico Garbe, Slavena Vylkova

**Affiliations:** Septomics Research Center, Friedrich Schiller University and Leibniz Institute for Natural Product Research and Infection Biology–Hans Knöll Institute, Jena, Germany; University of Maryland, Baltimore, UNITED STATES

## Introduction

The ability of fungi to colonize and persist within the human host is accompanied by an adaptation of fungal metabolism that allows them to withstand stress conditions, contend with the immune response, acquire nutrients, or simply secure a competitive edge during infection. Metabolites are the end products of cellular functions, and their levels reflect the fungal response to genetic or environmental changes. Despite the importance of metabolism for fungal fitness and pathogenicity, a comprehensive understanding of its impact on host-fungal interactions is still missing. Metabolomics, defined here as the simultaneous identification and quantification of the complete set of metabolites in a biological specimen, not only represents the chemical phenotype of an organism but also allows identification and interpretation of associations between genotype and phenotype. Investigations of the unique metabolic fingerprints of pathogenic microorganisms and the infection-associated changes to the host’s metabolism can provide a more complete impression of the infection process. Yet the utilization of metabolomics approaches to study host-fungal interactions are still few and far between. In this Pearl, we present an overview of the metabolome analyses in human pathogenic fungi to date, give examples of new discoveries made by such approaches, and discuss future research directions.

## What methods are available to study metabolomics?

**Fig 1:**
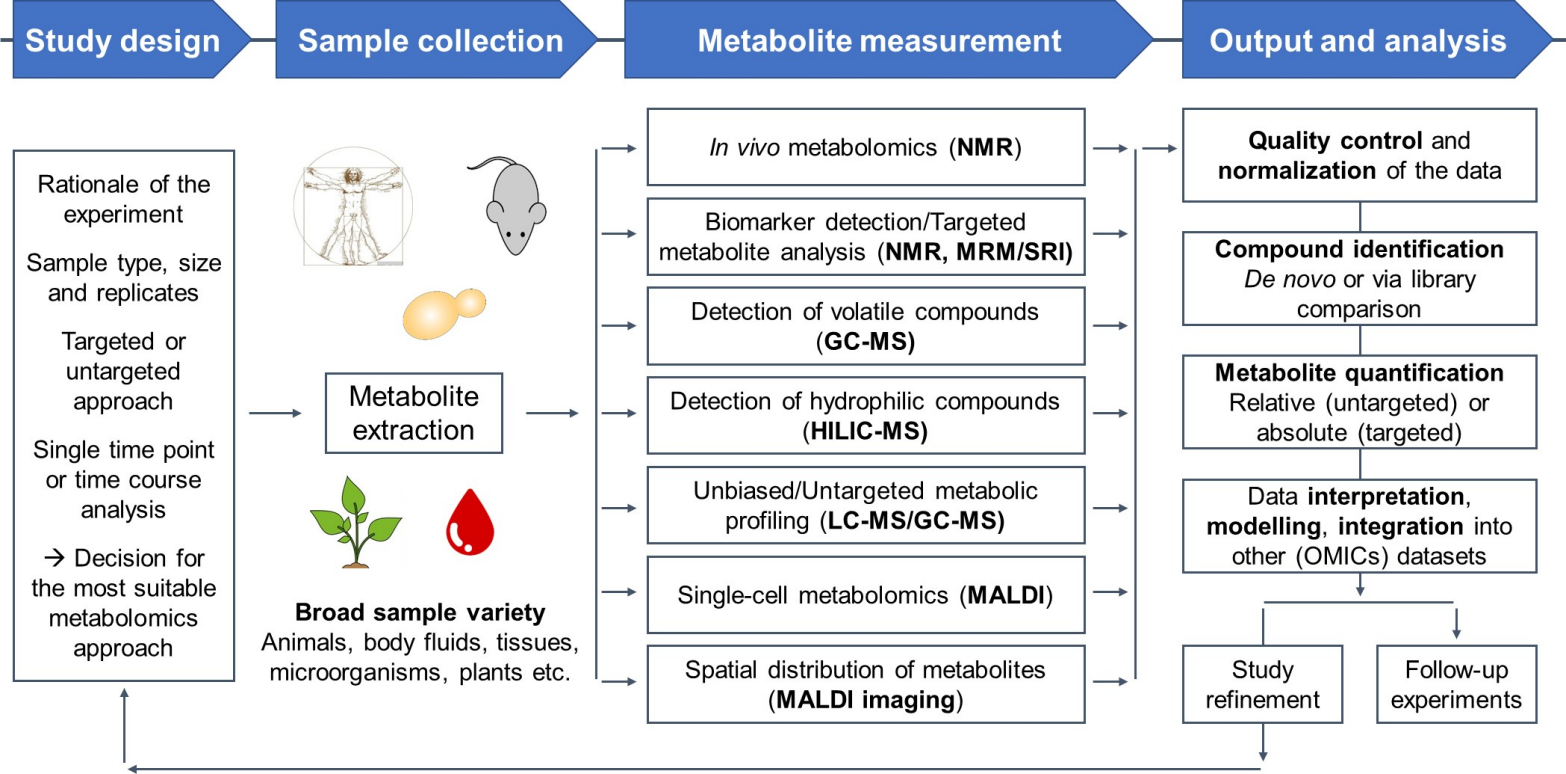
Overview of metabolomic approaches and typical workflow. The method of choice is shaped by the rationale of the experiment, the amount and type of the sample material (single or mixed cellular samples, tissues, supernatants/fluids, etc.), the decision for single measurements versus time course analyses, and whether the focus is on known or unknown (global) metabolic pathways/cellular processes (targeted versus untargeted approach, respectively). NMR is frequently used for the detection of biomarkers. Although this method provides high reproducibility and the option of in vivo metabolomics, it is less sensitive compared to MS techniques. Commonly used MS-based methods are MRM and SRI, usually performed with tandem MS. Typically, prior to MS measurements, a separation of the extracted metabolites is needed to increase sensitivity and specificity. Sample separation is done by either GC, more favorable for volatile compounds, or LC. HILIC is an LC technique optimized for separation of hydrophilic polar compounds, like carbohydrates. MALDI is a surface-based MS approach used in single-cell metabolomics, and for the determination of the spatial distribution of metabolites within a specimen via MALDI imaging. However, MALDI-based approaches are limited to abundant metabolites. The raw data processing includes method-dependent quality controls, as well as normalization and identification steps. The absolute quantification of the measured metabolites is only possible in targeted approaches. Finally, the processed data can be compared to existing databases, used for modeling of metabolic fluxes, or integrated with other OMICs datasets. GC, gas chromatography; HILIC, hydrophilic interaction chromatography; LC, liquid chromatography; MALDI, matrix-associated laser desorption ionization; MRM, multiple reaction monitoring; MS, mass spectrometry; NMR, nuclear magnetic resonance; SRI, selective reagent ionization.

Mass spectrometry (MS) and nucleic magnetic resonance (NMR) are the analytical tools of choice in most metabolomic studies, with a plethora of substrate- and study-specific variations available [1–3]. An overview of the most commonly used techniques and critical points to consider during study design is presented in Fig 1. Such approaches have been utilized in fungal research to perform untargeted monitoring of primary and secondary metabolites (SMs), targeted search for biomarkers (e.g., lipidomics), and in vivo measurement of metabolic fluxes [4–7]. For downstream analysis of metabolomic data, an increasing number of databases, software, and tools are available (summarized in [8, 9]).

## How to get the most out of your metabolomics data?

### Metabolomic databases and mathematical models

The required combination of analytical methods, complex data interpretation, and limited number of relevant studies in pathogenic fungi are the main reasons why researchers may hesitate to undertake metabolomic studies. However, the field is rapidly expanding due to the implementation of new methods, streamlined data analyses, and lower experimental costs. For instance, several fungal-specific metabolomic databases were recently launched, such as A2MDB, a comprehensive *Aspergillus fumigatus* SM repository [10]. A2MDB contains catalogued and annotated experimental metabolomics data, information about metabolic pathways, and molecular docking models of metabolite–protein target interactions. YMDB (http://www.ymdb.ca) is a database compiling information about metabolites found in or produced by *Saccharomyces cerevisiae*, which could be applied toward understanding metabolic responses of pathogenic yeasts (e.g., *Candida glabrata*). Another database collected curated literature information for 2,240 metabolic reactions in *Aspergillus niger*. The data were used to generate a mathematical metabolic model that was further validated with experimentally obtained transcriptional and metabolomic data [11]. Such databases and models are invaluable tools for making predictions and evaluating results, which increases the approachability of metabolomics research in pathogenic fungi.

### Integrated OMICs approaches

A few studies have linked metabolomics with other OMICs data, expanding our understanding of biological functions in a way that was not possible in single-OMICs studies. For example, comparative genomic analysis of *Cryptococcus neoformans* var. *grubii* versus *C*. *neoformans* var. *neoformans* and *Cryptococcus gattii* revealed a genomic translocation that disrupts *TGR1*, a gene encoding a newly described protein involved in metabolism. Metabolomic and subsequent phenotypic analyses showed that the deletion of *TGR1* leads to an accumulation of intracellular trehalose that is critical for protection against a variety of host-associated stresses [12]. Burgain and colleagues evaluated *Candida albicans* growth under hypoxia and combined transcriptomics and metabolomics data to show that oxygen limitation stimulates lipid biosynthesis, resulting in structural rearrangements of the cell membrane [13]. Another study linked proteomic and metabolomic data to gain insight into the drug resistance mechanisms of the emerging fungal pathogen *Candida auris* [14]. These are just a few examples that highlight the importance of multi-OMICs platforms that comprehensively utilize systems biology to draw more accurate conclusions about biological processes.

## What have we learned from metabolomics of human pathogenic fungi to this date?

### Identification of biomarkers

One key biological application of metabolomics is the identification of disease signatures and biomarkers. The routine measurement of single molecules or a pattern of several molecules as a part of fungus-specific metabolite imprints is rather inexpensive and could be used in diagnosis and therapeutic monitoring. Several metabolites have already shown promising characteristics for improved early *Aspergillus* detection [4]. As an example, testing of serum levels of gliotoxin, an SM with immunosuppressive properties, has been applied in the clinics [15]. A study searching for novel aspergillosis-specific biomarkers compared the secreted metabolites from 30 strains of several pathogenic fungi and identified a novel *Aspergillus*-specific linear tetrapeptide named aspergitide [16]. Another work utilized metabolomic-based approaches to improve the taxonomical identification of common human fungal pathogens [17]. A total of 45 primary metabolites from *A*. *pallidofulvus*, *Fusarium oxysporum*, and *Geothrichum candidum* could clearly differentiate between the species. Further, Ahmed and colleagues developed sampling methods for volatile organic compounds (volatome) in *A*. *fumigatus* [5], which were enriched in pyrazines and terpene. Others used a similar approach to identify species-specific volatomes of several *Candida* spp. and found that the *C*. *albicans* volatome was marked by increased 3-methyl-2-butanone and styrene, a feature absent in the other species [18]. These studies could lead to the development of breath or blood-based tests for the detection of fungal infections.

An important and thus far understudied aspect of fungal metabolomics is the ex vivo and in vivo metabolic sampling of the pathogen and the host during infection. The limited number of such studies already has revealed novel virulence characteristics. For example, metabolic profiles from meningitis rat model of cryptococcosis showed increased amounts of lactate, citrate, and polyols (mannitol and glycerol) and a decrease of glucose in the central spinal fluid [19]. Others showed that infection of lung epithelial cells with *C*. *neoformans* increases the secretion of pantothenic acid, previously found to stimulate fungal growth [20]. The implementation of newly identified metabolites and patterns into diagnostics or therapy will depend on several critical aspects, including early detection, reliability, low invasiveness, and costs.

### Effect of antifungal agents on fungal metabolism

Another major aim of metabolic studies in pathogenic fungi is to gain deeper insight in the effect of antifungal agents. Metabolomics-based approaches have been used, for example, to examine the effects of fluconazole on *C*. *albicans* metabolism [21, 22]. The drug increased the abundance of central carbon metabolism intermediates (e.g., glucose-6-phosphate, phenylpyruvate, α-ketoglutarate), whereas intermediates of amino acid and purine metabolism were decreased [21]. Beside fluconazole, the effects of other antifungal agents on the metabolism of *C*. *albicans* [23–25] and other pathogenic fungi [26, 27] have also been investigated. Targeted metabolomic analyses of clinically relevant *Mucorales* species following exposure to sublethal concentrations of posaconazole revealed significant alterations in ergosterol biosynthesis compared to *A*. *fumigatus*, e.g., accumulation of the toxic sterol 14-methylergosta-8,24-diene-3,6-diol [26]. Ergosterol biosynthesis, together with glycolysis and inositol biosynthesis, were among the iron-dependent pathways affected by the loss of the *C*. *neoformans* iron regulatory protein Cir1 [27], critical for fungal virulence. The knowledge acquired about drug-specific metabolic signatures and effects can help target vulnerable spots amenable to therapeutic intervention in the fungus.

### Virulence traits

The expression of virulence traits in human pathogenic fungi is frequently accompanied by distinct changes in metabolism. For example, metabolomic analysis of *C*. *albicans* revealed that the transition from yeast to hyphae, a crucial virulence factor, is accompanied by impaired central carbon and nitrogen metabolism [28]. Unlike yeast, hyphal cells had low intracellular ATP levels, whereas the levels of aromatic amino acids, proline, and fatty acids were increased. In accordance, the quorum sensing molecules farnesol or phenylethyl alcohol that suppresses hyphae formation stimulated the central carbon and energy metabolism [29, 30]. Hyphal-inducing compounds, such as the monosaccharide *N*-Acetyl-D-glucosamine (GlcNAc), also affect *C*. *albicans* metabolism. GlcNAc-grown cells had low intracellular levels of amino acids compared to cells grown in glucose. This led to an amino acid starvation response, a known trigger of hyphal morphogenesis [31]. Another study revealed that hyphal growth requires a functional glutamate dehydrogenase, an enzyme that links amino acid metabolism with the tricarboxylic acid (TCA) cycle [32]. Further, treatment of *C*. *albicans* with mitochondrial inhibitors led to the suppression of hyphae formation, which correlated with changes in the redox state, decreased TCA cycle activity, and increased catabolism of fatty acids compared to nontreated cells [6]. A combination of transcriptomic and metabolomic approaches defined the importance of *C*. *albicans* Snf5, a subunit of the SWI/SNF chromatin remodeling complex, in controlling metabolic flexibility and fungal fitness specifically under hypoxia [33].

Central carbon metabolism was also found to play a critical role in other pathogenic fungi. Metabolomics was used to characterize the function of a newly identified gene, *HVA1*, in *C*. *neoformans*. The mutant strain had increased levels of phosphoenolpyruvate and decreased levels of 2-ketoglutarate relative to the wild type, suggestive of a block in the TCA cycle and lowered ATP production. Further investigations showed that *HVA1* coordinates cell fitness (and thus virulence), likely via regulation of cellular NADPH levels [34]. Thus, metabolomic approaches aided in an understanding of stimulus-driven phenotypes and the construction of a more detailed framework of host-pathogen interactions.

### Biofilm formation

Several fungal pathogens can form robust biofilms on biotic surfaces and medical devices, which is a major health issue due to their reduced antifungal susceptibility. Metabolomic analyses of different stages of *C*. *albicans* biofilm formation showed that mature biofilms are characterized by low TCA cycle and mitochondrial activity, whereas the intracellular levels of several amino acids and glycerol (cellular response to osmotic stress) are elevated [35]. Moreover, trehalose that accumulated in the first 24 h of biofilm formation was critical for resistance to the antifungal drug amphotericin B [35]. Thus, metabolomic approaches revealed that both conservation of energy and increased production of stress-protective metabolites contributes to the antifungal resistance of cells within a biofilm.

Host-associated biofilms occur mostly as multispecies entities, which show different virulence characteristics compared to single-species biofilms. Metabolomic analyses of *Staphylococcus aureus* and *C*. *albicans* mixed biofilms showed that the symbiotic coexistence of the two species is signified by the high abundance of sedoheptulose-7-phosphate, an intermediate of the pentose phosphate pathway [36]. By contrast, the antagonistic effect of *Proteus mirabilis* on *C*. *albicans* growth resulted in slower metabolism and energy consumption by the fungus within the mixed biofilm [37]. Thus, metabolomics has an enormous potential to define interspecies interactions, which might be difficult or even impossible to achieve with other approaches.

### Interspecies interactions within the host

Besides in biofilms, interspecies interactions take place en masse in the human body (e.g., in the gut). Both metabolic modifications of the microbiome following antibiotic treatment and infection-associated changes to the host gut metabolome involving fungi have been investigated [38, 39]. Specifically, *C*. *albicans*–colonized mice had minimal changes in the cecum metabolites compared to the untreated animals [38]. However, mice treated with the antibiotic cefoperazone showed significant alterations in the microbiome and metabolome [39]. Intestinal levels of metabolites that promote *C*. *albicans* growth and morphogenesis, including carbohydrates, sugar alcohols, and primary bile acids, were increased after treatment with this antibiotic, whereas the levels of growth-inhibiting carboxylic acids and secondary bile acids were decreased [39]. Thus, metabolomic-based approaches are valuable tools for understanding the complex interactions between microbes and their host.

## Conclusions and perspectives

In conclusion, metabolomics and the respective bioinformatics tools and databases are rapidly evolving and have the potential to reveal novel aspects of metabolic adaptations in fungal pathogens. In contrast to transcriptomics, which measures changes in gene expression that might lead to metabolic rearrangements, metabolomics reveals the most downstream effects of cellular activity. Therefore, this technique brings more concrete insights into metabolic regulation and adaptation to changing environments. However, since metabolomics provides only a snapshot of the organism’s physiological state at the moment of sampling, the examination of metabolic fluxes or a time course of sample collection should be considered to fully understand metabolic dynamics and reprograming.

**Fig 2:**
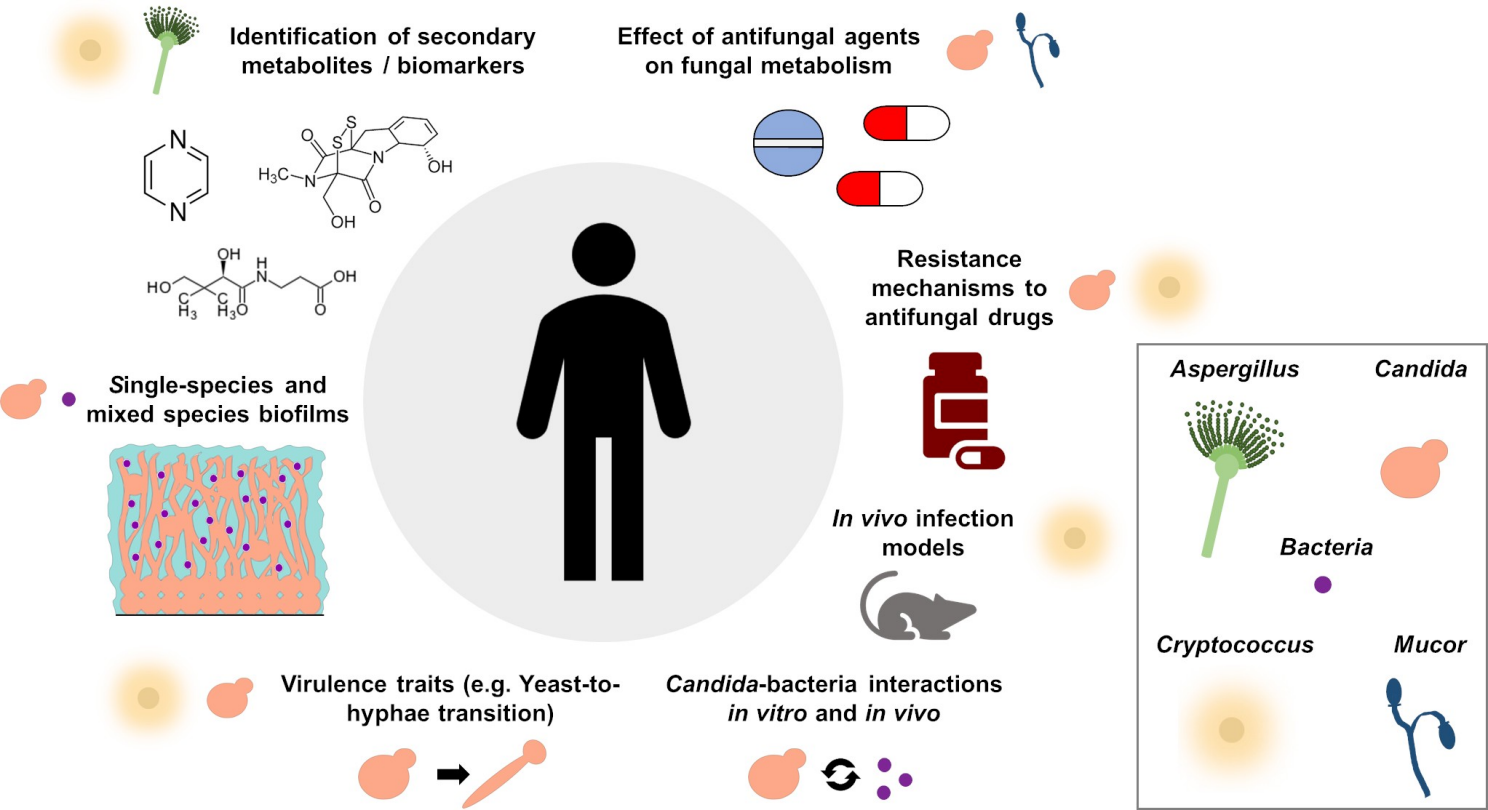
Metabolomics approaches in human pathogenic fungi. An overview of the metabolomics approaches utilized to date in human pathogenic fungi, with a primary focus on *Aspergillus*, *Candida*, *Cryptococcus*, and *Mucor* spp. In *Aspergillus*, metabolomic studies were used to identify secondary metabolites and biomarkers as a means to improve earlier detection and diagnosis of aspergillosis [4, 5, 10, 11, 15–17, 40, 41]. To date, metabolomics approaches have not been utilized to investigate the primary metabolism of *Aspergillus* spp., which has been the main focus of *C*. *albicans* and *C*. *neoformans* research. Metabolomic approaches brought insight into *C*. *albicans* virulence mechanisms, antifungal effects, and biofilm growth [6, 13, 18, 21–25, 28–33, 35–39]. Although one study focused on secreted metabolites and biomarkers in *C*. *auris* and used metabolomics to study resistance mechanisms in this organism [14, 43], metabolomics approaches in other pathogenic *Candida* spp. are still lacking. Further, metabolomic-based ex vivo, in vivo, and secondary metabolites/biomarker identification in *Candida* spp. are either scarce or lacking. In *Cryptococcus* spp. metabolomics was utilized in the identification of biomarkers, resistance mechanisms to antifungal drugs, and in vivo infection models [12, 19, 20, 27, 34]. In *Mucor* spp., the only metabolomic approach undertaken has examined the metabolic effect of antifungal agents [26].

In contrast to other well-established and widely used OMICs approaches, metabolomics is still a new tool. The studies performed in the field of fungal research to this date illustrate the broad applicability of the technique (Fig 2). However, several improvements are required for better utilization of this methodology, including optimization of the standard sampling and extraction protocols, the generation of user-friendly multi-OMICs databases, and mathematical prediction models of metabolic fluxes. For example, incorporating metabolomic datasets to established fungal resources, such as FungiDB, would improve data mining and interpretation. Further, new advances in the metabolomics field should be considered. For instance, Judge and colleagues used *Neurospora crassa* as a model organism for continuous in vivo monitoring of fungal metabolism [7]. In another study, antifungal drug activity was monitored in vivo and experimentally validated in *A*. *nidulans* [40]. Barkal and colleagues applied a micrometabolomic approach, in which an open microfluidic channel was used to collect SM from *A*. *fumigatus* incubated in culture media, blood, or coculture with bacteria [41]. Additionally, single-cell metabolomics was performed with *S*. *cerevisiae* to investigate potentially heterogeneous adaptations within a population to certain environmental conditions [42]. Moreover, matrix-associated laser desorption ionization (MALDI) imaging MS could be utilized to identify the spatial distribution of metabolites in a sample—for example, in infected patient tissue. Ultimately, there are many exciting possibilities in metabolomics research that can move the field of host-fungal interactions forward and toward an improvement of disease prevention and treatment.
